# Digital Transformation and Corporate Social Performance: How Do Board Independence and Institutional Ownership Matter?

**DOI:** 10.3389/fpsyg.2022.915583

**Published:** 2022-05-26

**Authors:** Shuang Meng, Huiwen Su, Jiajie Yu

**Affiliations:** ^1^School of International Trade and Economics, Central University of Finance and Economics, Beijing, China; ^2^Renmin Business School, Renmin University of China, Beijing, China; ^3^Business School, Beijing Normal University, Beijing, China

**Keywords:** digital transformation, corporate social responsibility, board independence, institutional ownership, corporate governance

## Abstract

This study addresses a gap in the literature on corporate governance and corporate social responsibility (CSR) by investigating whether and how board independence and institutional ownership moderate the relationship between digital transformation and corporate social performance (CSP). We find that digital transformation increases CSP using a panel dataset of Chinese publicly listed firms between 2014 and 2018. Moreover, we show that this positive impact is more pronounced when firms have higher proportions of independent directors on the board and institutional owners. These findings contribute to a better understanding of CSR dynamics, supporting the formulation and implementation of efficient CSR strategies in the digital era.

## Introduction

Over the past decade, the development of digital technologies, platforms, and infrastructures has reshaped all aspects of human life, with no sector or organization being immune. The digital revolution in the private sector, public institutions, and almost all industries has disrupted numerous markets, created new business opportunities, and fundamentally transformed business models (Bresciani et al., 2021; Kraus et al., 2021). These phenomena have increased research efforts in various streams of the business literature, such as marketing (Lamberton and Stephen, 2016; Kannan and Li, 2017), human resource management (Blanka et al., 2022; Fossen and Sorgner, 2022), innovation and entrepreneurship (Yoo et al., 2012; Nambisan et al., 2019; Usai et al., 2021), and business ethics and sustainability (Illia et al., 2017; Cardinali and De Giovanni, 2022; Schiavone et al., 2022). However, additional exploration seems needed.

The literature highlights the impact of digital transformation on firms’ performance, including financial (Kohtamäki et al., 2020; Chouaibi et al., 2022), international (Jin and Hurd, 2018; Li et al., 2018), and innovation performance (Bresciani et al., 2021; Usai et al., 2021). However, little attention has been devoted to its influence on corporate social performance (CSP), defined as the observable organizational outcomes of programs and policies intended to achieve corporate social responsibility (CSR) goals (Wood, 1991; Barnett, 2007; Orlitzky et al., 2017). Moreover, recent studies have investigated the use of digital technologies in green practices (Cardinali and De Giovanni, 2022), such as green innovation (Santoalha et al., 2021) and green supply chains (Ramirez-Peña et al., 2020). However, it is unclear whether a firm’s digital transformation enhances its overall CSP. On the one hand, CSR has become a critical item on top management’s agendas (The Economist, 2008; Aksoy et al., 2020). On the other hand, the drivers of CSP are of increasing relevance for practitioners and researchers (Brower and Mahajan, 2013; Orlitzky et al., 2017). Hence, we investigate whether and how a firm’s digital transformation significantly contributes to its CSP.

To examine the association between digital transformation and CSP, we apply stakeholder theory addressing a firm’s sensitivity to stakeholder needs, the diversity of stakeholder demands, and the exposure to stakeholder monitoring, as proposed by Brower and Mahajan (2013). Following previous studies (Hanelt et al., 2021; Verhoef et al., 2021; Blanka et al., 2022), we consider digital transformation as the adoption and application of digital technologies, such as artificial intelligence (AI), big data analytics (BDA), the Internet of Things (IoT), and information and communication technology to change business models and create or capture corporate value. Such a transformation involves fundamental changes in data acquisition, warehousing, analytics, and implementation (Kohtamäki et al., 2020; Paiola and Gebauer, 2020; Roblek et al., 2021; Vaska et al., 2021), affecting the way companies engage with stakeholders (Stohl et al., 2017; Dunn and Harness, 2019). Besides, digital transformation affects information sharing, exchange, and mutual monitoring between focal firms and their partners (Kouhizadeh and Sarkis, 2018; Cenamor et al., 2019; Ciampi et al., 2022). Hence, we argue that a higher level of digital transformation allows firms to better identify stakeholder expectations, increases the number and range of stakeholder needs, and strengthens firms’ monitoring intensity, thus improving CSP. We also argue that greater digital transformation enhances a firm’s ability to initiate and manage environmentally and socially responsible programs. Thus, we expect a positive relationship between digital transformation and CSP.

The literature has also mostly discarded the role that corporate governance mechanisms play in shaping firms’ digitalization, especially the use of digital technologies to achieve CSR goals. A large body of research (e.g., Crifo et al., 2019; Chen et al., 2020; Zaid et al., 2020; Bolourian et al., 2021) concentrates on the various dimensions of corporate governance for explaining the differences in CSP among firms. However, little is known about the interplay between corporate governance dimensions and digitalization, which affects a firm’s CSR attitudes and behaviors. Recent studies note that digital transformation entails changes in corporate structure, routine, culture, and future vision (Sousa and Rocha, 2019; Blanka et al., 2022), affecting existing business models (Bresciani et al., 2021; Verhoef et al., 2021). Hence, digitalization may be regarded as a crucial determinant of corporate governance. At the same time, digital transformation is a firm’s fundamental and strategic change directed by the board, initiated and implemented by the top management, and monitored by shareholders (Singh and Hess, 2017; Li et al., 2018). Therefore, effective corporate governance and digital transformation will interact, creating synergies between the firm’s decision-making and organizational outcomes, such as CSP.

Moreover, a firm’s ownership structure and its board structure, essential aspects of corporate governance (Jensen and Meckling, 1976; Fama and Jensen, 1983), may explain the notable differences in CSP across firms (Zaid et al., 2020; Beji et al., 2021). Various studies have shown that institutional investors and outside directors urge firms to integrate responsibility and sustainability into their operations (Dyck et al., 2019; García-Sánchez et al., 2019; Chen et al., 2020; Shahbaz et al., 2020). Accordingly, we posit that digital transformation is more likely to drive CSP in the presence of high board independence and institutional ownership levels. A test on a sample of publicly listed Chinese firms in 2014–2018 supports our research hypotheses.

We choose China as our empirical setting for two reasons. First, China has formulated a series of CSR laws and rules, such as regulations on environmental protection and information disclosure of listed companies, and has promoted voluntary CSR initiatives over the past two decades (Liao et al., 2018; Khan et al., 2021, 2022). Second, Chinese government has recently identified digitalization as one of the central parts of its public policy for catching-up with the advanced economies and Chinese firms have been encouraged to use digital mechanisms to transform their business (Lee et al., 2021; Lundvall and Rikap, 2022; Zhai et al., 2022). Therefore, China, as an aspirant economy (Bruton et al., 2021), provides an ideal context for studying our research questions.

The study’s contribution to the literature is threefold. First, it extends the current understanding of the antecedents of CSP by showing that digital transformation is an essential driver of CSP. We combine stakeholder theory and the literature about digitalization to highlight the mechanisms through which digital transformation determines CSP. Therefore, our study uncovers the crucial role of digital elements in the business ethics discussion. Second, we provide empirical evidence that a higher level of digital transformation increases overall CSP. Although previous research has investigated the influence of digital transformation on firm performance, it has largely ignored environmental and social performance. To the best of our knowledge, this study is one of the first attempts to examine the effect of digital transformation on CSP. Third, our study offers a novel theoretical contribution by investigating how corporate governance interacts with digital transformation in the CSP context. It is the first study to examine the moderating role of board independence and institutional ownership in the relationship between digital transformation and a firm’s CSP. In doing so, this study’s findings support the agency and stakeholder theories in the context of the current digital ecosystem.

## Theoretical Framework and Hypothesis Development

### Effect of Digital Transformation on CSP

Stakeholder theory essentially suggests that stakeholders’ preferences affect an organization’s decision-making and value creation in economic, social, environmental, and governance domains (Donaldson and Preston, 1995; Jones, 1995; Jones and Wicks, 1999). As Brower and Mahajan (2013) and Kang (2013) indicate, firms being more sensitive to their stakeholders’ needs, facing a wider range of stakeholder demands, or more closely scrutinized by multiple stakeholders tend to exhibit higher CSP. Accordingly, we posit that a firm’s digital transformation increases the salience and influence of stakeholders’ demands, thus increasing CSR participation and improving CSP.

First, digital transformation helps firms better communicate and interact with their customers and external partners, increasing sensitivity toward stakeholders’ expectations. For example, the use of big data and IoT allows firms to effectively and efficiently source, store, and share information regarding customers, suppliers, distributors, and other business actors (Kohtamäki et al., 2020; Paiola and Gebauer, 2020), and thus better responds to their needs and wants (Hadjielias et al., 2022; Schiavone et al., 2022). At the same time, the development of new algorithms and AI allows firms to parse, treat, and analyze a vast amount of data, extracting valuable information that helps identify stakeholders’ and societal needs (Cardinali and De Giovanni, 2022). The improved recognition of such needs triggers firms’ CSR engagement (Brower and Mahajan, 2013).

Second, digital transformation is positively related to the number and range of stakeholder demands and social issues that firms face. The use of digital media has significantly increased, changing the way firms communicate with stakeholders regarding CSR (Illia et al., 2017; Dunn and Harness, 2018; Vogler and Eisenegger, 2020). Using digital media (e.g., blogs, forums, and social media, among others), users create, circulate, and consume public information regarding focal firms’ products and services, called user-generated content (UGC). This allows them to quickly reach global audiences without having to pass through the gatekeeping function of media platforms (Kim and Johnson, 2016; Yang et al., 2019; Vogler and Eisenegger, 2020). In this vein, stakeholders may ask for firms’ CSR disclosures, question CSR initiatives, or voice skepticism about CSR efforts *via* social media (Stohl et al., 2017; Dunn and Harness, 2019). Hence, we argue that digital transformation motivates firms to focus on UGC, use various channels to engage in CSR communication, and maximize communication effectiveness (Illia et al., 2017). As a result, they are more likely to undertake CSR activities in response to large volumes and wide ranges of UGC about CSR.

Finally, digital transparency exposes firms to growing consumer, collaborator, and community pressures to increase environmental and social accountability. On the one hand, the application of digital technologies, such as blockchain and wireless broadband technologies, makes the origin and flow of information related to production, distribution, marketing, and consumption highly transparent, traceable, and available for consultation (Kouhizadeh and Sarkis, 2018; Ciampi et al., 2022). Thus, firms with a higher level of digital transformation may be subject to greater scrutiny and control from stakeholders and induced to engage in social performance initiatives (Gilliland et al., 2010; Orlitzky et al., 2017). On the other hand, with the spread of digital platforms such as cloud, e-commerce, and crowdfunding platforms, many firms are subject to mutual monitoring mechanisms (Cenamor et al., 2019). Hence, to collaborate with partners in the platforms, firms actively engage in CSR activities that reinforce stakeholder trust (Cuypers et al., 2015; Barnett, 2016) as an illegitimate act of one platform adopter is also detrimental to their partners’ reputations (Chou et al., 2017).

We also posit that a firm’s digital transformation improves its ability to initiate and manage CSR practices, improving CSP. First, digital technologies may increase combinatorial and radical green innovations (Ciarli et al., 2021; Santoalha et al., 2021). Thanks to digital technologies’ generative, modularized, and combinatorial properties, preexisting non-green technologies may be codified, combined, and recombined in emerging green domains, fostering unexpected recombinant green innovations (Quatraro and Scandura, 2019; Santoalha et al., 2021; Usai et al., 2021). Moreover, digital transformation may boost knowledge creation by changing a firm’s culture and employees’ mindset, ultimately supporting innovation (Sousa and Rocha, 2019). Second, digital technologies applied to production, transportation, supply chains, and recycling may reduce energy consumption, carbon emission, and industrial waste (Cardinali and De Giovanni, 2022). For example, by adopting automation and big data in shipbuilding, firms may predict faults and find welding solutions in advance, improving production efficiency and energy conservation (Ramirez-Peña et al., 2020). Cardinali and De Giovanni (2022) also suggest that Industry 4.0 technologies support circular economy systems, generating new opportunities for firms to improve CSR. Finally, digital applications help improve societal security (Newell and Marabelli, 2015; Vial, 2019). Due to its decentralized structure, blockchain technology may address concerns regarding information transparency and immutability and achieve supply chain sustainability, helping firms realize CSR goals (Kouhizadeh and Sarkis, 2018). Hence, we propose:

*H1*: Digital transformation is positively related to a firm’s CSP.

### Moderating Effect of Board Independence

Corporate governance affects companies’ CSR strategies and efforts and, thus, their CSP (Aguilera et al., 2007; Barnea and Rubin, 2010). Within corporate governance, company boards play a crucial role in the planning and decision-making process associated with CSR (Rao and Tilt, 2016; Crifo et al., 2019) and in the quality and reliability of CSR disclosure (Khan et al., 2013; García-Sánchez et al., 2019). There are various elements that signify board attributes, and board independence is one of the most important (Zaid et al., 2020; Bolourian et al., 2021). According to agency and stakeholder theories, a board with a larger proportion of independent directors (i.e., external or non-executive members) is more effective in supervising and controlling management (Dalton et al., 1999; Prado-Lorenzo and Garcia-Sanchez, 2010), ensuring that the social and environmental expectations of firms’ stakeholders are appropriately addressed (García-Sánchez et al., 2019). Further, non-executive directors, as outsiders compared to inside directors, safeguard the interests of company stakeholders because they are more likely to prevent opportunistic behaviors and enhance objectivity in the board’s decision-making (Fama and Jensen, 1983; Beasley, 1996). Therefore, we argue that boards with a higher proportion of independent directors are more likely to comply with stakeholder pressures arising from digital transformation and use digital technologies for addressing CSR issues, with a significant impact on CSP.

Previous studies have suggested that independent directors lean more toward environmental and social performance issues than internal directors (Ibrahim and Angelidis, 1995; Ibrahim et al., 2003). Being external to the organization, they receive less pressure from investors and managers, and their opinions are legally protected (Fuente et al., 2017; Liu et al., 2020). With greater independence and objectivity in their analysis of managerial decisions, non-executive directors focus more on satisfying the interests of firms’ stakeholders when pursuing economic goals. They tend to be more conscious of the need to improve the relationship between firms and their stakeholders (Liao et al., 2018; Shahbaz et al., 2020; Zaid et al., 2020). Therefore, a firm with a higher share of external members on the board is more likely to use digital technologies to identify stakeholders’ expectations and have a greater sensitivity to stakeholder needs when engaging in digital transformation.

Moreover, independent directors are more likely to acknowledge environmental and community concerns and be involved in CSR communication. Previous research (e.g., Fuente et al., 2017; Fernández-Gago et al., 2018; García-Sánchez et al., 2019) has observed that a more significant presence of independent directors on the board is related to a more extensive, transparent, and truthful CSR information disclosure. Furthermore, as independent directors generally support sustainable and ethical behaviors (Ibrahim et al., 2003), they are more willing to dialogue with different stakeholders on CSR issues. Hence, firms undergoing digital transformation are more likely to employ digital technologies (e.g., social media, forums, or micro-websites) to dialogue with stakeholders about CSR, respond to CSR demands, and show their commitment to social issues as the number of independent directors increases.

Finally, independent directors’ identity, prestige, and reputation seem closely connected with a firm’s CSR activities (Fernández-Gago et al., 2018). In this regard, leadership plays a crucial role in improving an organization’s environmental performance (Ahmad et al., 2021). Accordingly, boards with more non-executive directors tend to encourage their firms to behave in a more environmentally and socially responsible manner, refraining from negative media exposure and the subsequent loss of credibility (Johnson and Greening, 1999; Zaid et al., 2020). Since digital transformation increases monitoring by multiple stakeholders, independent directors will devote more effort to motivating companies undergoing digital transformation to invest in CSR activities in line with societal values. In addition, independent directors are more likely to stimulate firms to adopt digital technologies to achieve CSR goals, enhancing firms’ reputations and, in turn, their own reputation (Zaid et al., 2020). Thus, we predict:

*H2*: Board independence positively moderates the relationship between digital transformation and CSP such that higher levels of board independence strengthen the positive effect of digital transformation on CSP.

### Moderating Effect of Institutional Ownership

The ownership structure is another critical aspect of corporate governance (Fama and Jensen, 1983), which impacts firms’ CSR policies, procedures, and practices (Oh et al., 2011; Liu et al., 2019; Gao and Yang, 2021). Among multiple types of shareholders, an increasing number of institutional investors have committed to considering the environmental and social impacts of their investment decisions (Chen et al., 2020). With some exceptions (e.g., Brown et al., 2006; Cabral and Sasidharan, 2021), institutional investors (e.g., banks, investment companies, insurance companies, pension funds, and securities firms) tend to integrate sustainability and responsibility into their capital allocation choices, using ownership and monitoring attention to guide companies’ CSP (Graves and Waddock, 1994; Neubaum and Zahra, 2006; Oh et al., 2011; Dyck et al., 2019; Chen et al., 2020).

We argue that firms with a larger proportion of institutional owners are more sensitive and responsive to stakeholders’ needs and likely to invest in CSR activities when undergoing digital transformation. This is because institutional shareholders have additional incentives to detect and prevent opportunistic managerial behaviors and ensure stakeholders’ interests (Jensen and Meckling, 1976; Shleifer and Vishny, 1986). On the one hand, they can meet clients’ growing demands for sustainable investments by incorporating environmental and social responsibility factors into their portfolios and actively engaging in CSR issues with target companies (Dimson et al., 2015; Riedl and Smeets, 2017; Dyck et al., 2019). On the other hand, they can reduce the risks arising from investee firms’ negative externalities and signal their reliability and adherence to ethical values by improving the CSP of investee firms (Cox et al., 2004; Siegel and Vitaliano, 2007; Chen et al., 2020).

Further, institutional investors have more resources, expertise, and ability to monitor and impact firms’ strategic decisions (Oh et al., 2011; Ntim and Soobaroyen, 2013). These decisions include adopting digital technologies in green practices or other socially responsible programs (Cardinali and De Giovanni, 2022; Schiavone et al., 2022). In addition, since institutional investors are influential and cannot easily trade their shares (Shleifer and Vishny, 1986; Oh et al., 2011), they significantly influence firms’ strategic changes, such as digital transformation. At the same time, they tend to be more attentive to CSP, a long-term and essential determinant of sustainable organizational performance (Graves and Waddock, 1994). As a result, a higher level of institutional ownership may induce firms to integrate digital technologies within their CSR strategies since digitalization is not *per se* a socially responsible tool (Cardinali and De Giovanni, 2022; Fossen and Sorgner, 2022).

Responsible digital transformation, such as using BDA in green innovation and supply chains, may be viewed as a business model innovation (Bresciani et al., 2021; Hanelt et al., 2021; Cardinali and De Giovanni, 2022). We argue that responsible digitalization does not exclusively involve a firm’s resources and capabilities but may also be supported by institutional shareholders. First, institutional owners may help firms finance the resources they need for responsible digitalization and reduce the financial difficulties resulting from this choice to protect their investments against value erosion. Second, institutional investors have advantages in collecting information and engaging in active monitoring (Chen et al., 2007), which are needed to achieve firm innovation (Rong et al., 2017). Third, increased monitoring by institutional investors may shield managers from the reputational consequences of failed innovation (Aghion et al., 2013) and thus promote responsible digitalization. Consequently, we propose:

*H3*: Institutional ownership positively moderates the relationship between digital transformation and CSP such that higher levels of institutional ownership strengthen the positive effect of digital transformation on CSP.

**Figure 1:**
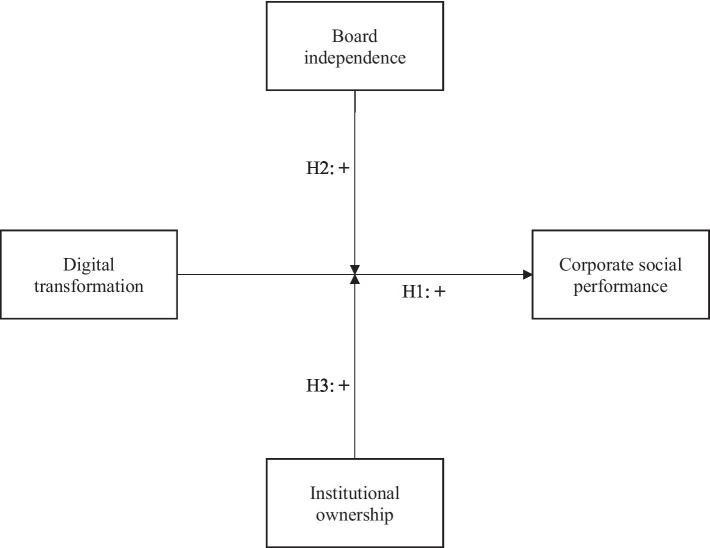
Conceptual framework.

Figure 1 summarizes the study’s theoretical framework.

## Methodology

### Data and Sample

This study uses a panel dataset of A-share firms listed on the Shanghai and Shenzhen stock exchanges from 2014 to 2018. We have obtained the data from the China Stock Market and Accounting Research Database (CSMAR) and Chinese Research Data Service Platform (CNRDS), which publish reliable information on listed firms’ financial and economic performance, shareholder backgrounds, details of the top management teams, and other related statistics. These data sources are widely used in research focusing on Chinese-listed firms (e.g., Jin et al., 2021; Meng and Sima, 2022; Zhu et al., 2022). We have obtained CSR data from Hexun, which publishes a CSR index for each listed firm every year. We exclude firms in the financial sector, observations with missing values, and unreliable data. The final sample is an unbalanced panel dataset comprising 2,281 firms and 10,048 firm-year observations.

### Measurement of the Variables

#### Dependent Variable

Following previous studies (Huang et al., 2018; Yang et al., 2021; Zhu et al., 2022), we measure firms’ *CSP* using Hexun’s CSR index, which measures firms’ CSP along five dimensions: shareholder equity responsibility; employee responsibility; supplier, customer, and consumer rights responsibility; environmental responsibility; and social responsibility. Previous studies commonly use these five dimensions to measure CSR (Cahan et al., 2017; Jin et al., 2021). We use the natural logarithms of the total score as the dependent variable in the empirical models.

#### Key Independent Variable

The key independent variable is the firm’s digital transformation (*DT*). Following previous studies (Hossnofsky and Junge, 2019; Gong and Ribiere, 2021; Yuan et al., 2021), we construct the digital transformation index by conducting a dictionary-based text analysis of the listed firms’ annual reports. The procedure is as follows. The first step identifies the keywords associated with digital transformation and establishes a dictionary of all variations of the keywords. The second step uses a machine-learning algorithm to obtain the frequency counts of those keywords from sample firms’ annual reports. The third and final step normalizes the frequency counts by the length of a specific section in the annual report (i.e., management discussion and analysis—MD&A) and computes the degree of digital transformation using the dictionary-based method. We construct the index of digital transformation using the CSMAR dataset.

#### Moderating Variables

The first moderator is the presence of an independent board. Following the previous research (Schnatterly and Johnson, 2014; Chen et al., 2016), we measure *board independence* as the percentage of independent members on the board who are also members of public organizations or professional institutions, academics, or investors with no direct ties with the firm.

The second moderator is institutional ownership. Following previous studies (Cahan et al., 2017; Harjoto et al., 2017), we measure *institutional ownership* as the percentage of a firm’s institutional holdings in the total shares outstanding at the end of the year, namely the sum of the shares of different institutional owners. In the CSMAR dataset, institutional owners are common funds, banks, qualified foreign institutional investor (QFII), insurance companies, brokers, security funds, entrust funds, financial companies, and other funding institutions.

#### Control Variables

Following previous research (Jin et al., 2021; Meng and Sima, 2022; Zhu et al., 2022), we control for various variables that may affect firms’ CSP. *Firm size* may influence CSP because larger firms have more resources to engage in CSR; we measure it as the natural logarithm of total assets. *Firm age* is calculated as the natural logarithm of the firm age, as previous studies indicate that older firms are less likely to favor social practice to gain legitimacy (Marquis and Qian, 2014). We control for return on assets (*ROA*) because profitability may affect firms’ CSR engagement and performance (Zhu et al., 2022). State ownership may also influence CSP because state-owned enterprises have stronger motivation to pursue and develop bonds with the government, making them more prone to engage in CSR engagement (Marquis and Qian, 2014); we measure it using a dummy variable (*SOE*) that equals 1 if the firm’s owner is the government, and 0 otherwise. We control for *leverage*, an indicator of financial risk, because a high level of leverage may hinder CSP (Liao et al., 2018); we measure it as the ratio of total debt to total assets. At the board level, we control for board size and CEO duality. We include *board size* in the models because larger boards are more inclined to engage in CSR (Jin et al., 2021); we measure it as the number of directors on the board. *CEO duality* is an indicator of executive power concentration and may affect firms’ CSR decisions (Khan et al., 2021); we measure it using a dummy variable that equals 1 if the CEO is also the board chair, and 0 otherwise. Finally, we measure *shareholder concentration* using the Herfindahl index of the share owned by the top-five shareholders, as previous studies indicate that ownership concentration affects the CSR decisions of the management team (Zhang et al., 2016). We winsorize the continuous variables at the 1 and 99% levels to eliminate the effects of extreme values. In addition, we include fixed effects in the models to control for the unobserved firm, industry, and city characteristics and common time trends. Table 1 reports the definitions of the variables.

**Table 1:** Variable definitions.

Variable	Definitions
CSP	The natural logarithm of the total score of corporate social performance
DT	The digital transformation index (please refer to the description above)
Board independence	The percentage of independent members on the board who belong to public organizations or professional institutions or are academics or investors with no direct ties with the firm
Institutional ownership	The ratio of institutional holdings of a firm to the total shares outstanding at the end of the year
Firm size	The natural logarithm of total assets
Firm age	The natural logarithm of firm age
ROA	Return on total assets
SOE	A dummy variable equal to 1 if the firm is an SOE, and 0 otherwise
Leverage	The ratio of total debt to total assets
Board size	The number of directors on the board
CEO duality	A dummy variable equal to 1 if the CEO is also the board chair, and 0 otherwise
Shareholder concentration	The Herfindahl index of the share owned by the top five shareholders

### Estimation Strategy

To test the proposed research hypotheses, we estimate the following models:


(1) $$CSP_{it}=\alpha +\beta \times DT_{it}+X_{it}\gamma +e_{i}+e_{j}+e_{c}+e_{t}+\varepsilon_{it}$$

(2) $$\begin{array}{c} CSP_{it}=\alpha +\beta \times DT_{it}+\delta \times digital_{it}\times \\ moderators_{it}+X_{it}\gamma +e_{i}+e_{j}+e_{c}+e_{t}+\varepsilon_{it} \end{array}$$

where *i* identifies a firm, *j* represents an industry, *c* denotes a city, and *t* is the year. The dependent variable $CSP_{it}$ is the logarithm of the CSR scores in the year. $DT_{it}$ is the firm’s digital transformation in the year, and $moderators_{it}$ are board independence and institutional ownership. $X_{it}$ is a vector of control variables. We denote firm fixed effects, industry fixed effects (two-digit industry), city fixed effects, and year fixed effects as $e_{i}$ , $e_{j}$ , $e_{c}$ , and $e_{t}$ , respectively; α is the intercept and $\varepsilon_{it}$ is the random error term. Model (1) is the baseline model for testing H1. Model (2) includes an interaction term to test for the moderating hypotheses. We cluster robust standard errors at the firm level to adjust for potential serial correlation and heteroskedasticity in all estimations.

## Results

### Descriptive Statistics and Correlation Analysis

Table 2 reports the descriptive statistics (mean and standard deviation) of the variables, allowing the exploration of the observed variations and supporting the proposed regression models. Table 3 presents the pairwise correlation matrix of all variables. We conduct variance inflation factor (VIF) analysis to address multicollinearity concerns. Table 2 also reports the VIF values of each variable, which range from 1.06 to 1.94, far below the conventional threshold value of 10. This evidence indicates that multicollinearity is not a severe concern in our models.

**Table 2:** Descriptive statistics.

Variable	Observations	Mean	Std. Dev.	VIF
CSP	10,048	2.970	0.758	
DT	10,048	0.275	0.371	1.07
Board independence	10,048	0.385	0.075	1.06
Institutional ownership	10,048	0.443	0.247	1.94
Firm size	10,048	22.283	1.301	1.81
Firm age	10,048	2.840	0.340	1.15
ROA	10,048	0.036	0.016	1.07
SOE	10,048	0.361	0.480	1.46
Leverage	10,048	0.425	0.201	1.55
Board size	10,048	10.265	2.628	1.14
CEO duality	10,048	0.263	0.440	1.13
Shareholder concentration	10,048	0.165	0.115	1.51

**Table 3:** Correlation matrix.

	Variables	(1)	(2)	(3)	(4)	(5)	(6)	(7)	(8)	(9)	(10)	(11)	(12)
(1)	CSP	1											
(2)	DT	0.111	1										
(3)	Board independence	0.021	0.063	1									
(4)	Institutional ownership	0.133	−0.198	−0.123	1								
(5)	Firm size	0.178	−0.141	−0.089	0.477	1							
(6)	Firm age	−0.010	−0.103	−0.130	0.145	0.175	1						
(7)	ROA	0.140	−0.006	0.034	−0.024	−0.137	−0.085	1					
(8)	SOE	0.014	−0.168	−0.147	0.435	0.392	0.273	−0.078	1				
(9)	Leverage	−0.127	−0.155	−0.068	0.263	0.554	0.193	−0.233	0.309	1			
(10)	Board size	−0.006	−0.088	−0.155	0.217	0.254	0.112	−0.017	0.264	0.174	1		
(11)	CEO duality	0.003	0.094	0.127	−0.222	−0.190	−0.133	0.054	−0.284	−0.135	−0.162	1	
(12)	Shareholder concentration	0.135	−0.142	0.026	0.538	0.256	−0.075	−0.022	0.212	0.076	0.032	−0.039	1

### Baseline Results

Table 4 presents the baseline regression results based on Equation (1). Model (1) only includes the control variables; Model (2) adds the key independent variable; and Model (3) includes the two moderators. The coefficient on digital transformation is significant and positive at the 5% level in Model (2) (coefficient = 0.121, *p* = 0.012), supporting H1, namely that digital transformation is positively related to a firm’s CSP. The coefficients on digital transformation are consistent across Models (2) and (3).

**Table 4:** Baseline regressions: digital transformation and CSP.

Model	(1)	(2)	(3)
Variables	CSP		
	Controls only	H1	H1
Firm size	0.311^***^	0.306^***^	0.295^***^
	(0.062)	(0.063)	(0.069)
Firm age	−0.245	−0.259	−0.250
	(0.221)	(0.220)	(0.216)
ROA	7.000^***^	7.057^***^	7.025^***^
	(0.774)	(0.774)	(0.784)
SOE	−0.107	−0.106	−0.107
	(0.127)	(0.126)	(0.125)
Leverage	−1.663^***^	−1.661^***^	−1.633^***^
	(0.107)	(0.108)	(0.114)
Board size	−0.012^***^	−0.012^***^	−0.014^***^
	(0.003)	(0.003)	(0.003)
CEO duality	0.020	0.020	0.019
	(0.053)	(0.053)	(0.053)
Shareholder concentration	1.038^***^	1.049^***^	0.911^**^
	(0.281)	(0.279)	(0.342)
DT		0.121^**^	0.123^**^
		(0.044)	(0.044)
Board independence			0.190^**^
			(0.087)
Institutional ownership			0.206
			(0.169)
Constant	−2.819^*^	−2.707	−2.638
	(1.607)	(1.628)	(1.698)
*N*	10,048	10,048	10,048
adj. *R*^2^	0.384	0.384	0.384

Robust standard errors in parentheses, ^*^p < 0.10, ^**^p < 0.05, and ^***^p < 0.01.

Table 5 presents the results of the moderation regressions based on Equation (2). Model (1) includes the interaction between digital transformation and board independence to test H2. Model (2) includes the interaction between digital transformation and institutional ownership to test H3. Model (3) includes all variables and interactions.

**Table 5:** Moderating effect regressions.

Model	(1)	(2)	(3)
Variables	CSP		
	H2	H3	Full model
Firm size	0.292^***^	0.297^***^	0.297^***^
	(0.068)	(0.070)	(0.070)
Firm age	−0.389	−0.238	−0.236
	(0.227)	(0.214)	(0.215)
ROA	6.332^***^	6.977^***^	6.946^***^
	(0.781)	(0.781)	(0.781)
SOE	−0.094	−0.104	−0.105
	(0.121)	(0.124)	(0.123)
Leverage	−1.692^***^	−1.633^***^	−1.634^***^
	(0.118)	(0.113)	(0.113)
Board size	−0.014^***^	−0.014^***^	−0.014^***^
	(0.003)	(0.003)	(0.003)
CEO duality	0.024	0.019	0.020
	(0.054)	(0.053)	(0.053)
Shareholder concentration	0.934^**^	0.940^**^	0.940^**^
	(0.338)	(0.351)	(0.351)
DT	0.080	0.014	0.152
	(0.086)	(0.042)	(0.090)
Board independence	0.059	0.190^**^	0.078
	(0.114)	(0.087)	(0.120)
Institutional ownership	0.241	0.125	0.120
	(0.177)	(0.182)	(0.183)
DT × board independence	0.470^**^		0.410^*^
	(0.228)		(0.223)
DT × institutional ownership		0.306^***^	0.324^***^
		(0.091)	(0.096)
Constant	−1.850	−2.681	−2.649
	(1.658)	(1.692)	(1.695)
*N*	10,048	10,048	10,048
adj. *R*^2^	0.375	0.385	0.385

Robust standard errors in parentheses, ^*^p < 0.10, ^**^p < 0.05, and ^***^p < 0.01.

**Figure 2:**
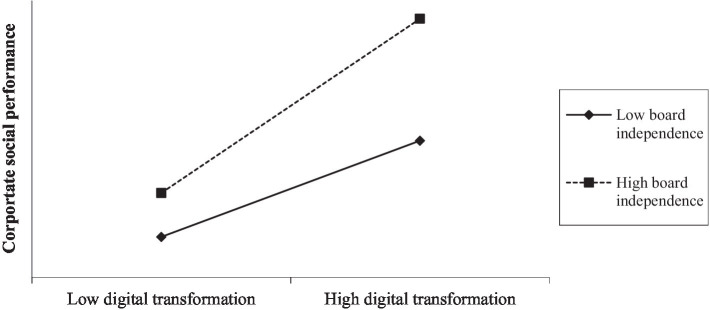
Moderating effect of board independence.

In Model (1) of Table 5, the coefficient on the interaction between digital transformation and board independence is positive and significant at the 5% level (coefficient = 0.470, *p* = 0.040). To better illustrate the results, we plot the interaction effects of board independence in Figure 2. Overall, the results support H2, indicating that board independence positively moderates the relationship between digital transformation and CSP.

**Figure 3:**
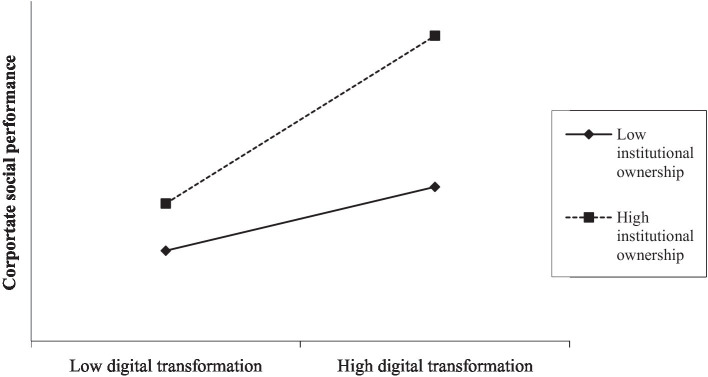
Moderating effect of institutional ownership.

In Model (2) of Table 5, the coefficient on the interaction between digital transformation and institutional ownership is positive and significant at the 1% level (coefficient = 0.306, *p* = 0.003). We plot the results in Figure 3. The evidence supports H3, namely, that institutional ownership positively moderates the relationship between digital transformation and CSP. The results of Model (3) are consistent with Models (1) and (2).

Regarding the control variables, firm size, ROA, and shareholder concentration have positive and significant effects on CSP, while board size has a negative and significant impact on CSP, mostly consistent with previous studies (e.g., Jin et al., 2021; Zhu et al., 2022). Overall, our results suggest that firms’ digital transformation positively impacts their CSP and that board independence and institutional ownership positively moderate the relationship between digital transformation and CSP.

### Robustness Checks

We perform several tests to confirm the robustness of our findings. We first perform regression analyses using alternative measures for the key variables. Then, we use the instrumental variable (IV) two-stage least squares (2SLS) approach to alleviate the endogeneity concern in the research design.

#### Alternative Measures for the Key Variables

First, we build an alternative proxy for CSP using the China Corporate Social Responsibility database published by the CNRDS, which has collected Chinese-listed firms’ CSP data since 2006 (Jin et al., 2021; Meng and Sima, 2022). The index covers six dimensions of CSR, namely employee relations, corporate governance, diversity, environment, product, and community, similar to the globally used CSR database KLD. The alternative measure for CSP is *CCSP*, calculated following Jin et al. (2021). However, the database used for analysis includes limited CSR information and only covers approximately 20% of listed firms. (Thus, we only use this data source for the robustness checks.) Table 6 reports the robustness test results, which are consistent with the baseline results.

**Table 6:** Robustness check: Alternative proxy for CSP.

Model	(1)	(2)	(3)
Variables	*CCSP*		
Firm size	0.253^***^	0.244^***^	0.239^***^
	(0.018)	(0.032)	(0.019)
Firm age	−0.003	0.182^*^	−0.024
	(0.064)	(0.087)	(0.064)
ROA	6.347^***^	5.553^**^	5.728^***^
	(1.356)	(2.232)	(1.369)
SOE	−0.046	−0.099	−0.070
	(0.044)	(0.074)	(0.045)
Leverage	−0.371^***^	−0.477	−0.362^***^
	(0.124)	(0.335)	(0.123)
Board size	0.003	−0.004	0.002
	(0.007)	(0.013)	(0.007)
CEO duality	−0.082	−0.113^***^	−0.066
	(0.053)	(0.030)	(0.053)
Shareholder concentration	0.161	0.044	−0.118
	(0.172)	(0.360)	(0.217)
DT	0.123^**^	0.078	0.056
	(0.055)	(0.206)	(0.110)
Board independence		0.377	0.036
		(0.339)	(0.259)
Institutional ownership		0.372^*^	0.201
		(0.195)	(0.154)
DT × board independence		0.330^*^	
		(0.168)	
DT × institutional ownership			0.479^**^
			(0.231)
Constant	−3.681^***^	−3.824^***^	−3.310^***^
	(0.436)	(0.755)	(0.460)
*N*	2,138	2,138	2,138
adj. *R*^2^	0.355	0.356	0.359

Robust standard errors in parentheses, ^*^p < 0.10, ^**^p < 0.05, and ^***^p < 0.01.

Second, instead of using the ratio of identified word counts to the total words in MD&A, we build an alternative proxy (*DTT*) for the independent variable using the total number of identified words related to digital transformation. Results in Table 7 indicate that they align with our main findings.

**Table 7:** Robustness check: Alternative proxy for digital transformation.

Model	(1)	(2)	(3)
Variables	CSP		
Firm size	0.202^***^	0.290^**^	0.196^***^
	(0.014)	(0.072)	(0.010)
Firm age	0.029	−0.269^*^	0.027
	(0.035)	(0.097)	(0.030)
ROA	6.830^***^	6.903^**^	6.767^***^
	(0.575)	(1.843)	(0.822)
SOE	−0.048^*^	−0.106	−0.058^**^
	(0.024)	(0.162)	(0.026)
Leverage	−1.245^***^	−1.628^***^	−1.249^***^
	(0.068)	(0.229)	(0.067)
Board size	−0.010^**^	−0.013	−0.010^***^
	(0.005)	(0.007)	(0.003)
CEO duality	0.021	0.017	0.026
	(0.023)	(0.056)	(0.021)
Shareholder concentration	0.512^***^	0.887^***^	0.412^***^
	(0.104)	(0.192)	(0.093)
DTT	0.081^**^	0.006	0.033
	(0.032)	(0.048)	(0.048)
Board independence		0.098	0.063
		(0.206)	(0.105)
Institutional ownership		0.205	0.008
		(0.102)	(0.057)
DTT × board independence		0.272^**^	
		(0.126)	
DTT × institutional ownership			0.329^***^
			(0.097)
Constant	−1.340^***^	−2.436	−1.203^***^
	(0.264)	(1.677)	(0.234)
*N*	10,048	10,048	10,048
adj. *R*^2^	0.298	0.383	0.388

Robust standard errors in parentheses, ^*^p < 0.10, ^**^p < 0.05, and ^***^p < 0.01.

#### Endogeneity

Reverse causality may exist between digital transformation and CSP. Therefore, we use the 2SLS approach to address this problem (Hossnofsky and Junge, 2019; Meng and Sima, 2022). Following previous studies (Gupta et al., 2018; Hossnofsky and Junge, 2019), we use the average digital transformation per industry as the instrument, ensuring that it is highly correlated with the firms’ digital transformation and is exogenous to the CSP. We use the “ivreghdfe” command in Stata to perform the 2SLS analysis. First, we need to confirm the instrument’s validity by performing various tests. The results show that it passes the exogeneity and identification tests. Table 8 reports the results of the second-stage 2SLS model; they are consistent with the baseline regression results, indicating that endogeneity is not a relevant concern in this study.

**Table 8:** Robustness check: Second stage of 2SLS estimations.

Model	(1)	(2)	(3)
Variables	CSP		
Firm size	0.148^***^	−0.390	−0.081
	(0.052)	(0.522)	(0.069)
Firm age	0.203^***^	0.209^***^	0.210^***^
	(0.007)	(0.007)	(0.007)
ROA	0.028	0.069^***^	0.076^***^
	(0.023)	(0.023)	(0.023)
SOE	6.919^***^	4.699^***^	4.679^***^
	(0.718)	(0.486)	(0.484)
Leverage	−0.047^**^	−0.077^***^	−0.075^***^
	(0.019)	(0.018)	(0.018)
Board size	−1.245^***^	−1.101^***^	−1.102^***^
	(0.046)	(0.044)	(0.044)
CEO duality	−0.010^***^	−0.013^***^	−0.013^***^
	(0.003)	(0.003)	(0.003)
Shareholder concentration	0.021	0.027	0.028
	(0.017)	(0.017)	(0.017)
DT	0.522^***^	0.398^***^	0.426^***^
	(0.068)	(0.075)	(0.075)
Board independence		0.183	0.131
		(0.368)	(0.096)
Institutional ownership		0.128^***^	0.018
		(0.040)	(0.060)
DT × board independence		2.108^*^	
		(1.182)	
DT × institutional ownership			0.350^**^
			(0.141)
*N*	10,048	10,048	10,048
adj. *R*^2^	0.195	0.225	0.227

Robust standard errors in parentheses, ^*^p < 0.10, ^**^p < 0.05, and ^***^p < 0.01.

## Discussion

### Theoretical Implications

This study makes three contributions to the literature. First, it enhances the current understanding of the antecedents of CSP by investigating the effect of firm-level digital transformation on CSP and exploring the underlying mechanisms from a stakeholder theory perspective. Previous studies have mainly documented the adoption of digital technologies in a specific CSR-related activity (Ramirez-Peña et al., 2020; Santoalha et al., 2021; Cardinali and De Giovanni, 2022). They have not examined the impact of organizational digitalization on its overall CSP. Our findings highlight the relevance of digital transformation in shaping a firm’s CSR behaviors and, hence, CSP. This study’s contribution is vital for future business ethics research, as digitalization is an essential driver of CSP. The integration between stakeholder theory and the literature on digitalization may offer novel perspectives to examine firms’ CSR strategies in the digital era.

Second, this is one of the first empirical studies to examine the effect of digital transformation on firms’ CSP. Previous research has mainly focused on the impact of digital transformation on financial, innovation, and internationalization performance (Jin and Hurd, 2018; Bresciani et al., 2021; Chouaibi et al., 2022). In contrast, firms’ social and environmental performance has been largely neglected. We offer evidence that digital transformation improves a firm’s CSP, supporting the assumptions of stakeholder theory (Brower and Mahajan, 2013).

Third, we provide new insights into how digital transformation and different corporate governance dimensions interact and influence CSP. To the best of our knowledge, this is the first study to investigate the interaction between digital transformation and corporate governance (i.e., board independence and institutional ownership). This study shows that as the proportion of independent directors on the board or the percentage of institutional owners increases, the relationship between digital transformation and firms’ CSP strengthens. Hence, we provide additional evidence that firms, managers, investors, and directors in the digital ecosystem behave in ways consistent with agency and stakeholder theories (Dyck et al., 2019; García-Sánchez et al., 2019; Zaid et al., 2020).

### Practical Implications

This study discusses how firm-level digital transformation may increase social gains and how corporate governance shapes their relationship. Therefore, it provides meaningful implications for business managers and policymakers.

In terms of management implications, this study emphasizes the importance of reacting to the new challenges of the digital era. CSR is of great value in an emerging economy such as China. Managers should exploit the positive externalities of digital transformation, increasing productivity and efficiency, with substantial social gains. Besides, firms should acknowledge the importance of their board composition and the impact of institutional ownership to encourage engagement in CSR.

Regarding policy implications, the government should implement coherent policies to foster the effective use of digital technologies and accelerate firms’ digital transformation. Coping with the challenges of digital transformation requires coordination across all policy domains. Building a comprehensive approach to digital transformation is vital. In addition, the government is fundamental to increasing awareness and building capacities for improving CSR among firms and stakeholders. Therefore, the government should foster better CSR practices using a multifaceted approach.

## Conclusion

Digital economy has contributed increasingly to economic development worldwide. Digital transformation is a widely observed phenomenon in the context of the current digital ecosystem. Thus, it is crucial to explore the impacts of digital transformation on business activities. Meanwhile, the role of corporate governance is important for firms’ strategy and sustainable development. However, the literature mostly neglects the role of corporate governance when studying the social impacts of firms’ digital transformation. By integrating stakeholder theory and the literature on digitalization, this study examines how digital transformation affects firms’ CSP. The study also develops a new conceptual framework to explain how corporate governance shapes the relationship between digital transformation and CSP, considering the moderating role of board independence and institutional ownership. In the empirical analysis, this study employs panel data estimation model and uses a dataset of 2,281 listed firms with 10,048 firm-year observations in China from 2014 to 2018 to test the hypotheses. To summarize, this study finds that digital transformation promotes firms’ CSP. In terms of corporate governance, it shows that higher board independence and institutional ownership levels strengthen the positive effect of digital transformation on CSP. Note that our findings are robust to a broad set of robustness analyses. These findings contribute to a better understanding of CSP in the context of emerging markets, which would also provide implications for the formulation and implementation of efficient CSR strategies in the digital era.

## Limitations and Future Research Direction

Although this study considerably contributes to the extant literature, some limitations can be addressed in future research. First, our sample is focused on listed firms since the information about their digital transformation and CSP is publicly available. Future research may well benefit from comparing the digital transformation processes in listed and non-listed firms that affect their CSR behavior. This is because non-listed firms may incur relatively weaker oversight and monitoring from government, media and other stakeholders. Second, it is possible that our research findings may not be completely generalized to other countries given the cross-country differences in institutional, economic and social–cultural conditions. Thus, future studies could test in different contexts to check whether the findings can be extended to other emerging or developed economies. Finally, we adopt a broad measure of digital transformation based on firms’ annual reports. Future research might complement our research by investigating specific components of digital transformation (e.g., digital marketing, digital servitization, digital culture, or digital process management).

## Data Availability Statement

The raw data supporting the conclusions of this article will be made available by the authors, without undue reservation.

## Author Contributions

SM performed conceptualization, methodology, visualization, and writing-original draft. HS did conceptualization, methodology, validation, and writing-original draft. JY was involved in conceptualization, methodology, validation, and writing-review and editing. All authors contributed to the article and approved the submitted version.

## Funding

This work was supported by the Fundamental Research Funds for the Central Universities and the Humanities and Social Science Fund of Ministry of Education of China (Grant No. 20YJC790099).

## Conflict of Interest

The authors declare that the research was conducted in the absence of any commercial or financial relationships that could be construed as a potential conflict of interest.

## Publisher’s Note

All claims expressed in this article are solely those of the authors and do not necessarily represent those of their affiliated organizations, or those of the publisher, the editors and the reviewers. Any product that may be evaluated in this article, or claim that may be made by its manufacturer, is not guaranteed or endorsed by the publisher.
